# Prognostic Value of the Three-Dimensional Right Ventricular Ejection Fraction in Patients With Asymptomatic Aortic Stenosis

**DOI:** 10.3389/fcvm.2021.795016

**Published:** 2021-12-13

**Authors:** Yosuke Nabeshima, Tetsuji Kitano, Masaaki Takeuchi

**Affiliations:** ^1^Second Department of Internal Medicine, School of Medicine, University of Occupational and Environmental Health, Kitakyushu, Japan; ^2^Department of Cardiology and Nephrology, Wakamatsu Hospital of University of Occupational and Environmental Health, Kitakyushu, Japan; ^3^Department of Laboratory and Transfusion Medicine, School of Medicine, Hospital of University of Occupational and Environmental Health, Kitakyushu, Japan

**Keywords:** asymptomatic aortic stenosis, prognosis, 3DE, RVEF, CART analysis

## Abstract

**Background:** The right ventricular (RV) function is an important prognostic marker of asymptomatic aortic stenosis (AS). However, previous publications have not addressed the additive value of conventional RV parameters over left heart parameters. Whether three-dimensional echocardiography (3DE)-derived RV ejection fraction (RVEF) has prognostic utility independent of 3DE derived left heart parameters is also unknown. We investigated the prognostic utility of 3DE RVEF in patients with asymptomatic AS.

**Methods:** We retrospectively selected 392 asymptomatic AS patients. RVEF, left ventricular ejection fraction (LVEF) and left atrial volumes (LAVs) were measured using 3DE datasets. We determined the association of those parameters, as well as of aortic valve replacement (AVR), and Charlson's comorbidity index with cardiac events. We also analyzed whether RVEF has incremental value over two-dimensional echocardiography (2DE) RV parameters.

**Results:** During a median follow-up of 27 months, 57 patients developed cardiac events, and 68 patients received AVR. Univariate Cox proportional hazard analysis revealed that RVEF was associated with cardiac events (*p* < 0.001). Multivariate analysis revealed that RVEF was significantly associated with cardiac events (*p* < 0.001) even after adjusting for AVR, Charlson's comorbidity index, LVEF, LAV, E/e', and indexed aortic valve area (iAVA). An incremental value of RVEF over left heart parameters was also demonstrated using a nested regression model. Classification and regression-tree analysis selected RVEF first with a cut-off value of 41%. RVEF had incremental value over iAVA, LVEF, and 2DE conventional RV parameters for its association with future outcomes.

**Conclusions:** 3DE RVEF had significant prognostic value even after adjusting for comorbidities, left heart parameters, and conventional 2DE RV parameters in asymptomatic aortic stenosis.

## Introduction

The number of patients with aortic stenosis (AS) has been increasing rapidly due to the aging of society, especially in developed countries (1). This change has resulted in an exponential increase of heart failure (HF) hospitalizations and surgical or transcatheter aortic valve replacements (AVRs) (2). Due to the socioeconomic impact of AS, optimization of surgical intervention is urgently required. Current guidelines recommend AVR for patients with symptomatic severe AS (3). However, management of asymptomatic AS with preserved left ventricular ejection fraction (LVEF) has not been clarified (3–6). When considering AVR in asymptomatic AS patients (6, 7), we have to assess not only valve condition, but also myocardial function, because of the non-negligible rate of sudden cardiac death and relatively higher risk of HF (8–10). The prognostic impact of right ventricular (RV) function has garnered increased interest in this parameter in recent years. RV dysfunction can affect the risk of HF via a negative impact on cardiac output and ventricular interactions in various heart diseases (11). Genereux and colleagues have proposed a new concept called “cardiac damage stage” for risk stratification of asymptomatic AS patients (12, 13). According to their findings, patients with RV dysfunction had worse prognoses than those with left ventricular (LV) dysfunction with preserved RV function. Galli et al. also indicated the importance of tricuspid annular plane systolic excursion (TAPSE) to predict cardiovascular death in severe AS (14). However, those publications used 2D or Doppler echocardiography to analyze RV function, irrespective of complex RV geometry. Three-dimensional echocardiography (3DE) does not rely on geometric assumptions and can provide more accurate and reliable information regarding RV function (15). Left atrial (LA) function is also reported as a useful marker to stratify the risk of asymptomatic AS patients (16). We hypothesized that RV ejection fraction (RVEF) is the most robust predictor for future outcomes among LV, LA, and RV function parameters assessed with 3DE in asymptomatic AS. Accordingly, we sought to investigate the prognostic utility of 3DE-derived RVEF in patients with asymptomatic AS.

## Materials and Methods

### Study Population

This was a single-center observational study. Using a 3DE database, we retrospectively selected 3DE datasets of Japanese patients with AS who had undergone transthoracic echocardiography in the University of Occupational and Environmental Health hospital from April 2008 to December 2018. Individuals who had no AS-related symptoms were selected from the database. Patients with fewer than 30 days of follow-up after echocardiography were excluded. We also collected several clinical parameters to calculate Charlson's comorbidity index (17). The study was approved by the ethics committee at the University of Occupational and Environmental Health. As this was a retrospective study, the Institutional Review Board waved the requirement for informed consent.

### Echocardiography

3DE was performed immediately after standard transthoracic 2D echocardiography (2DE) and Doppler echocardiography. 3DE images were acquired using an apical approach and commercially available ultrasound machines (iE33 or Epic7G, Philips Healthcare, Andover, MA; Vivid7 or Vivid E95, GE Healthcare, Horten, Norway) equipped with a 3DE transducer (X3-1 or X5-1, Philips Healthcare, Andover, MA; 4V, GE Healthcare, Horten, Norway). Trans-mitral flow velocity was recorded at the coaptation point of both leaflets. Mitral annular velocities were recorded at septal and lateral sides of the mitral annulus, and average e' was calculated. Peak aortic flow velocity was recorded in multiple transducer positions, and the highest value was used for the measurements of the mean pressure gradient (PG) and velocity-time integral (VTI). RV fractional area change (RVFAC) was calculated by standard formula. 2DE RV speckle tracking analysis was performed using commercially available, vendor-independent, fully automated strain analysis software (AutoStrain RV, TomTec Imaging Systems, Unterschleissheim, Germany). The software automatically determined the RV endocardial border and performed speckle tracking analysis during a single cardiac cycle. The endocardial border was manually corrected as required. RV free-wall longitudinal strain (RVfwLS) and global longitudinal strain (RVGLS) were calculated.

### 3D Speckle Tracking Analysis

**Figure 1:**
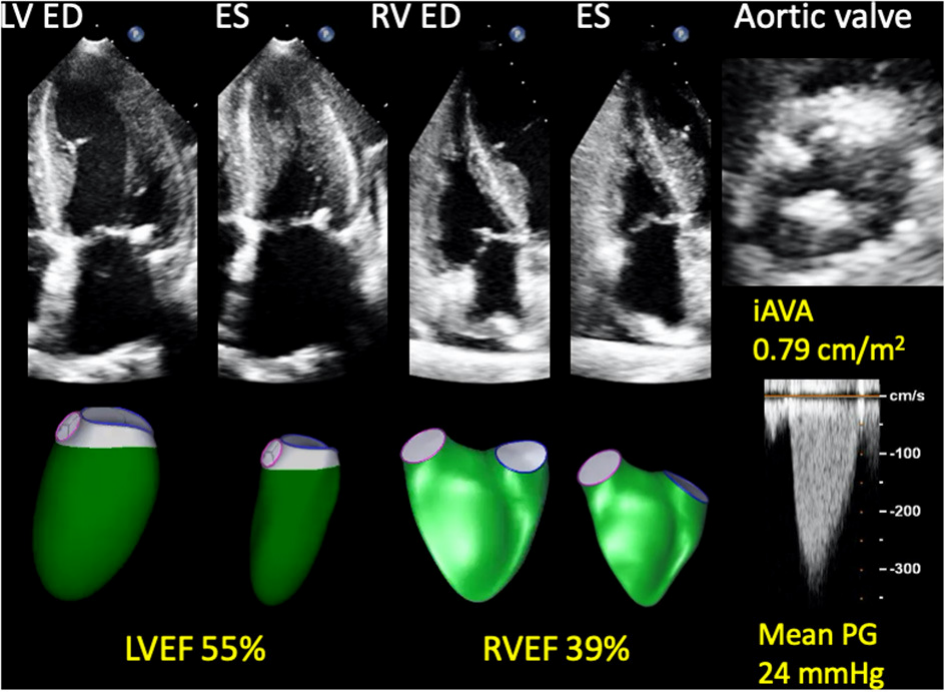
Representative 3D RV and LV analyses in a patient with asymptomatic AS who developed a cardiac event. A 74-year-old woman who developed heart failure three months after baseline echocardiography. AS was moderate and LVEF was preserved; however, RVEF was reduced. She also had hypertension, chronic kidney disease, and chronic obstructive pulmonary disease. 3D, three-dimensional; AS, aortic stenosis; ED, end-diastole; ES, end-systole; iAVA, indexed aortic valve area; LV, left ventricular; LVEF, left ventricular ejection fraction; PG, pressure gradient; RV, right ventricular; RVEF, right ventricular ejection fraction.

LV and RV 3DE speckle tracking analyses were performed using commercially available, vendor-independent, 3DE quantification software (4D LV ANALYSIS-3 and 4D RV FUNCTION 3, TomTec Imaging Systems). These methods have been described in detail previously, and their accuracy and reliability are recognized (18). For LV analysis, after selecting a specific 3DE dataset, the software automatically detected the LV endocardial border. Manual correction of the endocardial border was performed as needed. After confirming the tracing line, 3D speckle-tracking analysis was performed through a single cardiac cycle, generating LV volume curves, from which LV end-diastolic volume (LVEDV), end-systolic volume (LVESV), and LVEF were calculated. LV mass was calculated by manually drawing the epicardium of end-diastolic frames of three standard apical views extracted from 3DE datasets. LVEDV index (LVEDVI), LVESV index (LVESVI), and LV mass index (LVMI) were calculated by body surface area (BSA). For the RV analysis, LV-focused end-diastolic apical four-, two-, and three-chamber views, and RV-focused two-orthogonal views were extracted from a 3DE dataset. After specifying anatomical landmarks (center points of the mitral and tricuspid annular planes and the apex), the software automatically determined the RV endocardial cast. Following manual correction of the endocardial border, 3D speckle tracking analysis was performed. RV end-diastolic volume index (RVEDVI), end-systolic volume index (RVESVI), and RVEF were calculated (Figure 1).

### 3D Left Atrial Volume

3D maximal and minimal left atrial volumes (LAVs) were calculated using vendor-specific software (QLAB 13.0, Philips Healthcare, Andover, MA; EchoPAC version 203, GE Healthcare, Horten, Norway). Detailed methods have been described previously (19). Briefly, we calculated LAVs using Simpson's biplane method with the anterior-posterior and medial-lateral 2D views extracted from 3DE datasets. LAVs were indexed to BSA yielding maximal LAV index (LAVIx) and minimal LAV index (LAVIn). This is a manual method, and we did not use 3D LA speckle tracking software.

### AS Severity

Stroke volume (SV) was calculated as the difference of LVEDV and LVESV measured by 3DE. Stroke volume index (SVi) was calculated as SV divided by BSA. Indexed aortic valve area (iAVA) was calculated as SV divided by AV VTI times BSA. We defined more-than-moderate-to-severe AS as AVA <1.0 cm^2^ or iAVA <0.6 cm^2^/m^2^. The patients were classified as high-gradient (peak velocity ≥4.0 m/s or mean PG ≥40 mmHg) AS (HG-AS), and low-gradient (peak velocity <4.0 m/s and mean PG <40 mmHg) AS (LG-AS) according to the current ESC guideline (20). LG-AS was further classified into 3 categories: low-flow (SVi <35 mL/m^2^) low-gradient AS (LFLG-AS) with preserved LVEF, LFLG-AS with reduced LVEF, and normal-flow low-gradient AS (NFLG-AS).

### Follow-Up

Patients were followed up in an outpatient clinic. Prognostic information was obtained by interviewing attending physicians or by searching digital medical records. If patients were followed up in other hospitals, we also contacted corresponding physicians to obtain prognostic information. The primary endpoint was defined as a composite adverse cardiac event, including cardiac death, hospitalization due to heart failure, ventricular tachycardia/fibrillation, or non-fatal myocardial infarction.

### Statistical Analysis

Continuous data were expressed as means ± SDs or as medians and interquartile ranges (IQR; 25th to 75th percentiles). Categorical variables were presented as absolute numbers or percentages. Student's *t*-test was used to compare continuous variables between two groups when data were normally distributed. Wilcoxon sum rank test was used when data were not normally distributed. Fisher's exact test or a chi-square test were used to compare categorical variables. Cox proportional hazards analysis was used to calculate hazard ratios and 95% confidence intervals. Univariate and multivariate Cox proportional hazard analyses were used to assess the prognostic utility of echocardiographic parameters. AVR was treated as a time-dependent covariate. Survival analysis was performed using the Kaplan-Meier method, and differences in survival rates between groups were analyzed by the log-rank test. Net-reclassification improvement (NRI) analysis and DeLong's test were used to compare risk prediction utility between the two models. Classification and regression-tree (CART) analyses were conducted to predict cardiac events. A two-sided *p*-value < 0.05 was considered statistically significant. All statistical analyses were performed using commercial software (JMP version 14.0, SAS Institute Inc., Cary, NC; R version 3.6.3, The R foundation for Statistical Computing, Vienna).

## Results

### Patient Characteristics

Using a 3DE database, we found 392 asymptomatic AS patients. Of those patients, 25 were excluded because their follow-up duration after baseline echocardiography was <30 days. Finally, first-time echocardiographic datasets from 367 patients were selected for the analysis. We could not analyze 3DE datasets of twelve patients due to a low volume rate or extremely poor image quality. We performed 3DE LV and RV analysis in the other 355 patients, resulting in feasibility of 97% for both LV and RV analyses. Image quality of LV was good in 35%, fair in 53%, and poor in 12%. Corresponding RV values were good in 13%, fair in 57%, and poor in 30%. It was impossible to analyze 3D LAVs due to the fact that datasets failed to encompass all of the LA wall in 10 patients, and the feasibility of 3D LA analysis was 94%. LA image quality was good in 35%, fair in 53%, and poor in 11%. Clinical characteristics are summarized in Table 1.

**Table 1:** Patient characteristics (*n* = 367).

**Variables**	
Age (y.o)	77.1 ± 10.0
Sex (Male)	168 (46%)
Body mass index (kg/m^2^)	22.7 ± 3.9
Body surface area (m^2^)	1.52 ± 0.21
Systolic blood pressure (mmHg)	147.5 ± 23.5
Diastolic blood pressure (mmHg)	75.0 ± 13.5
Heat Rate (bpm)	69.5 ± 12.6
Rhythm (SR/Af/PM)	338/25/4
**Comorbidities**	
Hypertension	295 (80%)
Diabetes mellitus	115 (31%)
Coronary artery disease	79 (22%)
Chronic kidney disease	174 (47%)
Chronic pulmonary disease	62 (17%)
**Medications**	
Calcium channel blocker	185 (52%)
β-blocker	76 (21%)
ACEi or ARB	208 (58%)
Digitalis	7 (2%)
Diuretics	107 (30%)
Warfarin/DOAC	58 (16%)
Charlson's index	5.22 ± 1.94

*Data are shown as mean ± standard deviations or n (%)*.

*ACEi, angiotensin converting enzyme inhibitor; Af, atrial fibrillation; ARB, angiotensin receptor blocker; DOAC, direct oral anticoagulant; SR, sinus rhythm; PM, pacemaker*.

### Two- and Three-Dimensional Echocardiographic Parameters

Severe AS was observed in 43% (152/354) of the study population. Median values of LVEF and RVEF by 3DE analysis were 52 and 48%, respectively. 30% (107/355) of patients had reduced LVEF (LVEF <50%), and 32% (112/355) had reduced RVEF (RVEF <45%). 53% (189/355) of patients had preserved both ventricular EFs, and 15% (53/355) of patients had reduced bilateral ventricular EFs. Table 2 presents echocardiography parameters.

**Table 2:** Echocardiographic parameters (*n* = 367).

**Variables**	**Number**	**Median (25th to 75th percentiles)**
E-wave (cm/s)	367	77.0 (62.0–98.0)
E/A	335	0.71 (0.59–0.90)
E/e'	363	16.9 (12.8–22.1)
MR (severe/moderate/mild)	318	3/5/219
TR (severe/moderate/mild)	274	3/12/218
SPAP (mmHg)	326	33.0 (27.9–39.2)
PH (SPAP > 50 mmHg)	326	20 (6%)
RVFAC (%)	342	40.1 (36.1–44.8)
RVfwLS (%)	342	24.6 (20.7–27.8)
RVGLS (%)	342	19.4 (16.6–21.8)
Peak velocity (m/s)	366	3.04 (2.53–3.59)
Mean PG (mmHg)	366	19.4 (13.8–29.8)
Indexed AVA (cm^2^/m^2^)	354	0.64 (0.51–0.80)
AS classification	354	
HG-AS		44 (12%)
LFLG-AS with preserved LVEF		24 (7%)
LFLG-AS with reduced LVEF		21 (6%)
NFLG-AS		108 (30%)
Mild to moderate AS		158 (45%)
SVi (mL/m^2^)	355	42.5 (37.0–49.1)
3D LVEDVI (mL/m^2^)	355	83.3 (72.1–97.3)
3D LVESVI (mL/m^2^)	355	39.9 (33.1–49.2)
3D LVEF (%)	355	52.5 (47.9–55.6)
3D LVMI (g/m^2^)	355	98.8 (83.9–113.6)
3D LAVIx (mL/m^2^)	345	50.1 (39.7–61.3)
3D LAVIn (mL/m^2^)	345	26.9 (21.1–36.1)
3D RVEDVI (mL/m^2^)	355	55.9 (48.4–66.4)
3D RVESVI (mL/m^2^)	355	29.4 (24.2–34.1)
3D RVEF (1%)	355	48.0 (43.7–52.7)

*Data are shown as medians (25th to 75th percentiles) or n (%)*.

*3D, three-dimensional; AVA, aortic valve area; LAEF, left atrial emptying fraction; LAVIn, minimum left atrial volume index; LAVIx, maximum left atrial volume index; LVEDVI, left ventricular end-diastolic volume index; LVEF, left ventricular ejection fraction; LVESVI, left ventricular end-systolic volume index; LVMI, left ventricular mass index; MR, mitral regurgitation; PG, pressure gradient; PH, pulmonary hypertension; RVEDVI, right ventricular end-diastolic volume index; RVEF, right ventricular ejection fraction; RVESVI, right ventricular end-systolic volume index; RVFAC, right ventricular fractional area change; RVfwLS, right ventricular free-wall longitudinal strain; RVGLS, right ventricular global longitudinal strain; SPAP, systolic pulmonary artery pressure; SVi, stroke volume index; TR, tricuspid regurgitation*.

### Outcomes

During a median follow-up of 26.7 months (IQR, 15.4–56.6 months), 57 patients reached a primary endpoint, including 19 cardiac deaths, 32 HFs requiring hospital admission, four myocardial infarctions, and two ventricular tachyarrhythmias. Notably, four cardiac deaths and three HFs occurred after AVR. Sixty-eight patients received AVR during the follow-up. The median interval from baseline echocardiography to AVR was 20.5 months. Univariate Cox proportional hazard analysis revealed that mean PG, iAVA, E/e', SVi, Charlson's comorbidity index, LVEF, RVEF, and LAVIs were associated with cardiac events (Table 3).

**Table 3:** Univariate Cox regression analyses of predictors of cardiac events.

	**HR**	**95% CI**	***Z*** **-score**	***P*** **-value**
Age (per 1 y.o increase)	1.025	0.994–1.058	1.569	0.117
Sex (Male)	1.237	0.735–2.083	0.801	0.423
BMI (per 1 kg/m^2^ increase)	0.956	0.888–1.030	−1.181	0.238
BSA (per 1 m^2^ increase)	0.738	0.196–2.787	−0.448	0.654
SBP (per 1 mmHg increase)	0.993	0.982–1.004	−1.318	0.188
DBP (per 1mmHg increase)	0.996	0.977–1.015	−0.419	0.675
Heat Rate (per 1 bpm increase)	1.030	1.010–1.051	2.899	0.004
E-wave (per 1 cm/s increase)	1.010	1.003–1.017	2.809	0.005
E/A (per 1-unit increase)	1.298	0.808–2.087	1.078	0.281
E/e' (per 1-unit increase)	1.031	1.013–1.049	3.349	<0.001
MR (moderate or severe)	2.303	0.557–0.529	1.152	0.250
TR (moderate or severe)	2.301	0.700–7.569	1.372	0.170
SPAP (per 1 mmHg increase)	1.015	0.989–1.042	1.117	0.264
PH (yes)	2.874	1.222–6.757	2.420	0.016
RVFAC (per 1% increase)	0.916	0.884–0.949	−4.866	<0.001
RVfwLS (per 1% increase)	0.887	0.841–0.936	−4.399	<0.001
RVGLS (per 1% increase)	0.820	0.765–0.880	−5.535	<0.001
Peak velocity (per 1 m/s increase)	1.283	0.911–1.081	1.427	0.153
Mean PG (per 1 mmHg increase)	1.020	1.002–1.039	2.163	0.030
Indexed AVA (per 1 cm^2^/m^2^ increase)	0.065	0.013–0.317	−3.384	<0.001
SVi (per 1 mL/m^2^ increase)	0.945	0.916–0.975	−3.598	<0.001
AVR (yes)	0.539	0.272–1.335	−1.335	0.182
Charlson's index (per 1-point increase)	1.296	1.137–1.487	3.876	<0.001
3D LVEDVI (per 1 mL/m^2^ increase)	1.006	0.994–1.017	0.965	0.335
3D LVESVI (per 1 mL/m^2^ increase)	1.027	1.014–1.039	4.215	<0.001
3D LVEF (per 1% increase)	0.894	0.868–0.920	−7.559	<0.001
3D LVMI (per 1 g/m^2^ increase)	1.012	1.004–1.020	2.979	0.003
3D LAVIx (per 1 mL/m^2^ increase)	1.023	1.011–1.036	3.812	<0.001
3D LAVIn (per 1 mL/m^2^ increase)	1.034	1.022–1.046	5.508	<0.001
3D LAEF (per 1% increase)	0.941	0.921–0.960	−5.790	<0.001
3D RVEDVI (per 1 mL/m^2^ increase)	1.017	1.003–1.032	2.435	0.015
3D RVESVI (per 1 mL/m^2^ increase)	1.060	1.041–1.079	6.437	<0.001
3D RVEF (per 1% increase)	0.891	0.864–0.917	−7.669	<0.001

*3D, three-dimensional; AVA, aortic valve area; AVR, aortic valve replacement; BMI, body mass index; BSA, body surface area; CI, confidence interval; DBP, diastolic blood pressure; LAEF, left atrial emptying fraction; LAVIn, minimum left atrial volume index; LAVIx, maximum left atrial volume index; LVEDVI, left ventricular end-diastolic volume index; LVEF, left ventricular ejection fraction; LVESVI, left ventricular end-systolic volume index; LVMI, left ventricular mass index; HR, hazard ratio; PG, pressure gradient; RVEDVI, right ventricular end-diastolic volume index; RVEF, right ventricular ejection fraction; RVESVI, right ventricular end-systolic volume index; SBP, systolic blood pressure; SPAP, systolic pulmonary artery pressure; SVi, stroke volume index*.

**Figure 2:**
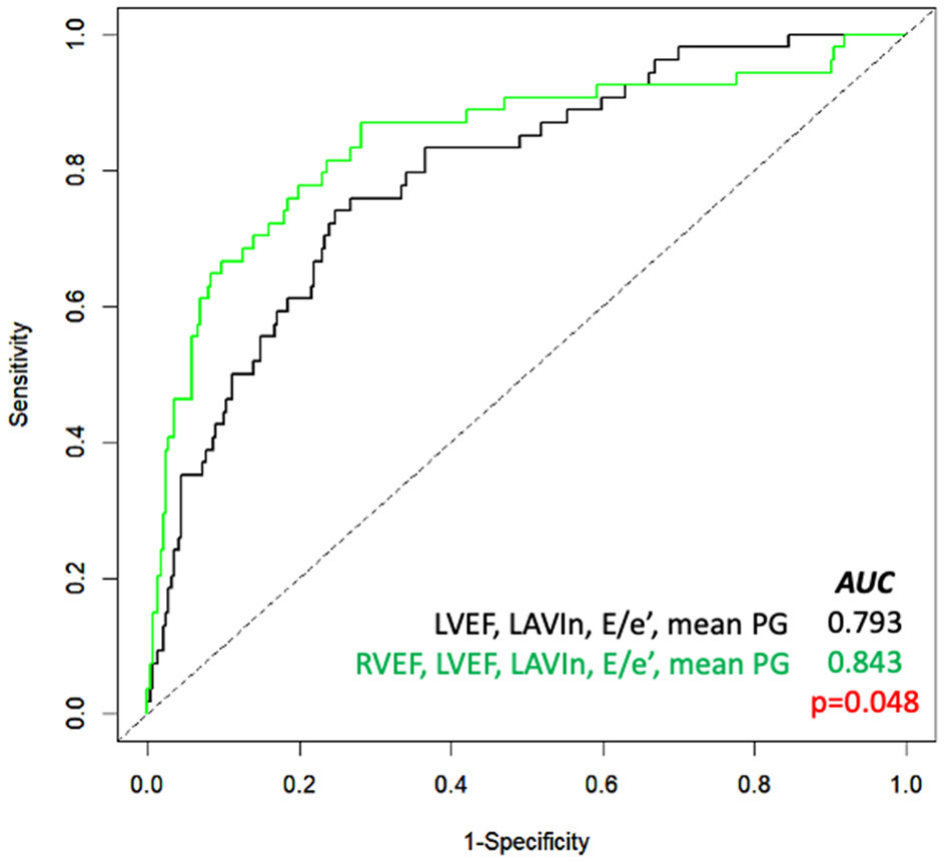
Receiver-operating characteristic curve analysis for prediction of cardiac events. Comparison of the area under the curve (AUC) of several parameters (black line) and with RVEF added (green line). LAVIn, minimum left atrial volume index; LVEF, left ventricular ejection fraction; PG, pressure gradient; RVEF, right ventricular ejection fraction.

Table 4 depicts the results of multivariate Cox proportional hazard analyses. According to results from univariate analysis and clinical importance, we created five models for multivariate analyses. LVEF and RVEF were significantly associated with cardiac events after adjusting Charlson's comorbidity index, AVR, and one of either echocardiographic parameter. DeLong's test revealed a significant increase of c-statistics after adding RVEF to LVEF, LAVIn, E/e', and mean PG (Figure 2). NRI analysis revealed that the logistic regression model that included RVEF showed a significant improvement of outcome classification compared with the logistic regression model including LVEF, LAVIn, E/e', and mean PG (NRI = 0.655; *p* < 0.001).

**Table 4:** Multivariate Cox regression analyses after adjusting Charlson index and AVR as time-dependent covariates.

	**Mean PG model**		**iAVA model**		**SVi model**		**E/e' model**		**LAVIn model**	
	**HR (95% CI)**	***P*** **-value**	**HR (95% CI)**	***P*** **-value**	**HR (95% CI)**	***P*** **-value**	**HR (95% CI)**	***P*** **-value**	**HR (95% CI)**	***P*** **-value**
LVEF	0.933 (0.900–0.968)	<0.001	0.944 (0.910–0.978)	0.002	0.945 (0.908–0.983)	0.005	0.951 (0.916–0.988)	0.010	0.954 (0.917–0.993)	0.021
RVEF	0.918 (0.887–0.949)	<0.001	0.924 (0.893–0.956)	<0.001	0.918 (0.884–0.954)	<0.001	0.913 (0.881–0.947)	<0.001	0.926 (0.892–0.961)	<0.001
Mean PG	1.038 (1.020–1.055)	<0.001								
iAVA			0.073 (0.015–0.357)	0.001						
SVi					0.999 (0.971–1.029)	0.981				
E/e'							1.035 (1.009–1.062)	0.009		
LAVIn									1.024 (1.008–1.039)	0.002

*AVR, aortic valve replacement; CI, confidence interval; HR, hazard ratio; iAVA, indexed aortic valve area; LAVIn, minimal left atrial volume index; LVEF, left ventricular ejection fraction; RVEF, right ventricular ejection fraction; SVi, stroke volume index*.

**Figure 3:**
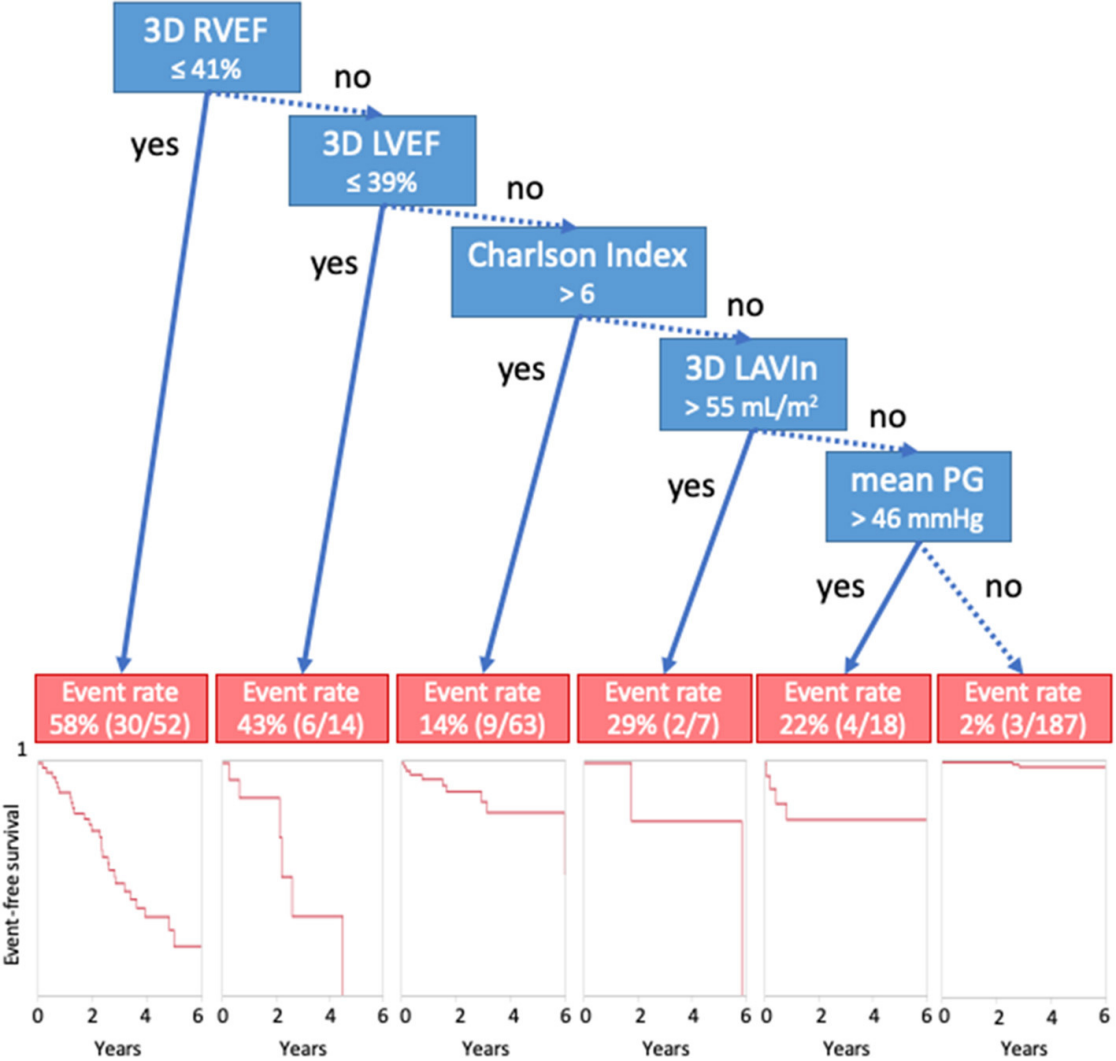
Classification and regression-tree analysis for cardiac events. Blue boxes denote cut-offs, and red boxes describe event rates. 3D, three-dimensional; AVR, aortic valve replacement; LAVIn, minimum left atrial volume index; LVEF, left ventricular ejection fraction; PG, pressure gradient; RVEF, right ventricular ejection fraction.

For survival CART analysis, we entered 18 variables including age, sex, Charlson's comorbidity index, LVEDVI, LVESVI, LVEF, LVMI, SVi, LAVIx, LAVIn, peak velocity, mean PG, iAVA, E wave, E/e', RVEDVI, RVESVI, and RVEF. CART first selected RVEF (with a cut-off of 41%), followed by LVEF (with a cut-off of 39%), Charlson's cormorbidity index, LAVIn, and mean PG (Figure 3). Generated Kaplan-Meier curves indicated that only 4% of patients (9/212) having more than cut-off values of LVEF, RVEF, and Charlson's comorbidity index ≤ 6 developed cardiac events. Corresponding values of patients with ≤ 41% of RVEF and ≤ 39% of LVEF were 58% (30/52) and 43% (6/14), respectively.

**Figure 4:**
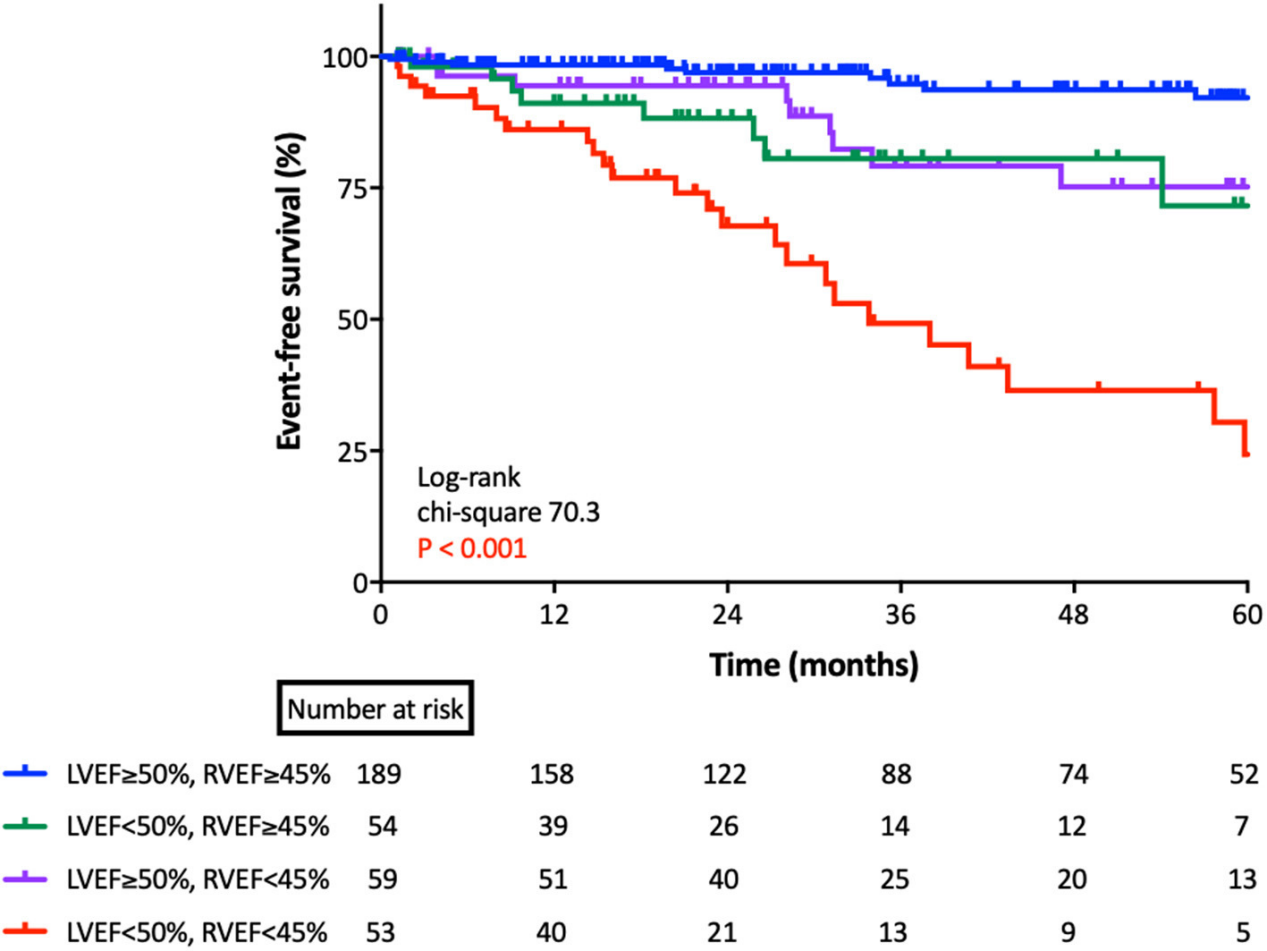
Kaplan-Meier survival analysis for cardiac events based upon RVEF and LVEF. Kaplan-Meier analysis for cardiac events, according to group, as classified by lower limits of normal LVEF and RVEF. LVEF, left ventricular ejection fraction; RVEF, right ventricular ejection fraction.

For the Kaplan-Meier analysis, we divided patients into four groups according to cut-off values of LVEF (50%) and RVEF (45%) (Figure 4). There was a significant difference between the group of patients who had preserved LVEF with impaired RVEF and the group of patients with both EFs preserved (*p* < 0.001).

### RVEF and Conventional 2D RV Parameters

RVGLS was significantly associated with cardiac events even after being adjusted for AVR, Charlson's comorbidity index, LVEF, and either mean PG, iAVA, E/e', LAVIn, or SVi (Supplementary Tables S1–S3). RVfwLS had no significant association with outcome when LAVIn was included in the multivariate model. However, when we added RVEF to the multivariate model, the prognostic significance of all of three 2D RV parameters disappeared (Supplementary Table S4). To determine the independent and incremental prognostic value of RVEF, we generated a nested regression model, sequentially adding iAVA, LVEF, conventional 2D RV parameters (either FAC of RVGLS), and RVEF. Chi-square values increased in stepwise fashion upon adding each parameter. Addition of RVEF to the model including iAVA, LVEF and RVFAC further increased the chi-square value from 57.7 to 71.7 (*p* < 0.001), whereas adding it to the model containing iAVA, LVEF and RVGLS increased it from 61.5 to 72.7 (*p* < 0.001), respectively (Supplementary Figure S1).

### Patients With Preserved LVEF

For sensitivity analysis, we performed subgroup analyses in a subset of patients who had ≥50% of LVEF. Among 248 patients with LVEF ≥50%, 23 patients developed cardiac events during a median of 32 months follow-up. Multivariate Cox analysis, revealed that RVEF was a significant marker even after adjusting for LVEF (*p* < 0.001). Supplementary Figure S2 shows the results of the CART analysis. As with the results for all patients, CART selected RVEF first.

### Patients With “More-Than-Moderate-to-Severe AS”

Among 196 “more-than-moderate-to-severe” AS patients, 40 developed cardiac events during a median of 30 months follow-up. Univariate Cox regression analysis indicated that RVEF and conventional 2DE RV parameters were significantly associated with cardiac events (Supplementary Table S5). The results of multivariate Cox regression analyses revealed that RVEF was significantly associated with cardiac events, even after adjusting for AVR, Charlson index, and LVEF / iAVA/ LVMI /E/e' /LAVIn (Supplementary Table S6). We constructed a nested regression model incorporating iAVA, LVEF, RVFAC or RVLS, and RVEF in stepwise manner to determine the independent and incremental prognostic value of RVEF. RVEF had a significant incremental value over iAVA, LVEF, and RVFAC (Supplementary Figure S3, left panel) and over iAVA, LVEF, and RVGLS (Supplementary Figure S3, right panel). Supplementary Figure S4 shows the CART analysis indicating that LVEF was selected first with a cut-off value of 47%, followed by RVESVI with a cut-off value of 39 mL/m^2^.

### Patients With “Less-Than-Moderate-to-Severe AS”

Among 158 “less-than-moderate-to-severe” AS patients, 16 developed cardiac events during a median of 28 months follow-up. Univariate Cox regression analysis revealed that RVEF and RVGLS were significantly associated with cardiac events (Supplementary Table S7). CART analysis selected RVEF at first with a cut-off value of 42% (Supplementary Figure S5).

### Factors Associated With Reduced RVEF

We performed logistic regression analysis to determine which parameter was associated with reduced RVEF, which was defined as RVEF <40%. Univariate logistic regression analysis revealed that heart rate, E/e', iAVA, SVi, LVEF, LAVIn, coronary artery disease (CAD), chronic kidney disease (CKD), and atrial fibrillation (AF) were significantly associated with reduced RVEF. Multivariate logistic regression analyses revealed that iAVA, SVi, LVEF, and LAVIn were significantly associated with reduced RVEF, even after adjusting for CAD, CKD, and AF (Supplementary Table S8).

## Discussion

To the best of our knowledge, this is the first study to demonstrate the prognostic utility of 3DE determined RVEF in patients with asymptomatic AS.

### Previous Studies

Indications and best timing of AVR for patients with asymptomatic AS are not clearly defined. Some studies suggest that early elective AVR improves the prognosis of patients with asymptomatic severe AS (21). Genereux et al. proposed the “cardiac damage” concept, which claims that damage to each heart chamber regulates the prognosis of patients with severe AS who received AVR, and their results suggest that when RV damage has occurred, it is too late to intervene (12). Moreover, Tastes et al. reported that the cardiac damage system has significant prognostic utility for patients with moderate to severe asymptomatic AS, independent of AVR (13). Because of its reduced invasiveness and lower perioperative risk, the advent of transcatheter AVR could increase the frequency of valve replacement in patients who are at high risk for surgical AVR. For this reason, pre-operative risk stratification of patients with asymptomatic AS is vital for optimal use of health resources.

Some reports have explained the prognostic utility of RV functional parameters in AS. Galli et al. reported the prognostic importance of RV function among 200 patients with severe AS (14). The subjects were predominantly asymptomatic (87%). They found that 24% of severe AS patients had impaired RV function, and biventricular dysfunction was a strong prognostic indicator of future cardiac events. Zilberszac et al. reported the prognostic value of RV dysfunction in 76 patients with low-flow, low-gradient severe AS (22). Only seven patients were asymptomatic. RV dysfunction was assessed by TAPSE, the systolic lateral tricuspid annulus velocity or 2D RV free-wall strain. RV dysfunction was a significant prognostic marker for overall survival in univariate Cox proportional hazard analysis. However, this utility was no longer significant after adjusting EuroSCORE II. Those two papers used only RV parameters that represent longitudinal motion. This limitation may explain why in those studies, RV parameters were not such strong markers compared with other factors, because the RV moves three-dimensionally, and regional and longitudinal motion may not represent global RV function in some cases (23).

### Current Study

We found that 3D RVEF had significant utility in predicting future cardiac events, even after adjusting for LVEF, AS severity, LV diastolic function, AVR, and comorbidities. Statistical analysis revealed that RVEF had incremental value over comorbidities, left heart parameters, and AS severity to predict cardiac events. Kaplan-Meier analysis indicated that both LVEF and RVEF were useful for risk stratification of patients with asymptomatic AS.

In our cohort, 73% of asymptomatic AS patients had some non-cardiac comorbidities. Not only AS severity and left heart function, but also other comorbidities that develop RV volume or pressure overload, such as pulmonary disease and CKD can affect RV function. Thus, RV function may represent status of the whole heart. In fact, reduced RVEF was significantly associated with echocardiographic parameters, CKD, CAD, and atrial fibrillation. We cannot determine the causal relationship because of the cross sectional nature of the study; however, optimal medical therapy or intervention targeting HF, ischemia, and AF have potential to improve management of AS patients with reduced RVEF.

Our results are consistent with previous publications. Previously, we determined the prognostic utility of 3D RVEF in 446 patients with diverse backgrounds (18). 3D RVEF had an incremental value over 3D LVEF, E/e', and CKD to predict future adverse events. CART analysis selected RVEF first with a cut-off of 41%. The value was equivalent to observed value in this study (Figure 3). Muraru et al. also reported the prognostic utility of 3D RVEF in patients with various cardiac diseases (24). They stressed that 3D RVEF showed higher predictive power for cardiac events than TAPSE or RVFAC, which was in agreement with our results. Kaplan-Meier analysis indicated that patients with RVEF <40% showed a high incidence of cardiac death, cardiac events, and all-cause mortality. Those results are consistent with our RVEF cut-off values derived from CART analysis. Unlike those two previous studies, our intent was to determine prognostic value of RVEF in a specific group of patients, asymptomatic AS, and we clearly showed its prognostic utility.

In a subset of patients with “more-than-moderate-to-severe” AS, CART analysis selected LVEF first. The current AHA/ACC guideline (3) recommends aortic valve surgery as class I indication for asymptomatic severe AS patients with LVEF <50%. Therefore, we believe that assessing LVEF at first is reasonable in asymptomatic severe AS. CART secondly selected RVESVI. However, we must note that 7 of 28 more-than-moderate-to-severe AS patients with LVEF ≥50%, who also had RVEF <45% developed cardiac events. Of those 7 patients who had cardiac events, two developed heart failure after AVR. Thus, when we manage asymptomatic severe AS patients, we should assess LV function for indications of AVR first. However, we should carefully manage patients with preserved LVEF, but impaired RVEF. On the other hand, for patients with asymptomatic mild/moderate AS, the prognostic importance of RVEF may be greater than that of left heart function and valve condition.

### Clinical Implications

Assessment of RVEF using 3DE provides robust information for predicting future outcomes in patients with asymptomatic AS. Overall, 3D RVEF was significantly associated with cardiac events regardless of AS severity or left chamber function. 3DE determined that RVEF is a much more useful parameter than 2DE-derived RV parameters for predicting future outcome. Thus, combined 3DE assessment of left and right chamber function might be the best way for appropriate management of patients with asymptomatic AS.

## Limitations

This study has several limitations. First, it includes not only severe AS patients, but also mild to moderate AS patients. However, because AS is a progressive disease, predicting future outcomes of patients with mild to moderate AS using baseline echocardiography is essential for appropriate management. In fact, RV systolic functions were significant prognostic parameters, independent of AS severity. Second, 3D LAV analysis was based on manual tracing, even though 3D LV and RV analyses were performed using semi-automated 3D speckle tracking software. This difference could underrepresent the prognostic value of LAVs in this study, and further studies using 3D LA speckle tracking analysis are required. Third, we did not include 3D global strain values. It is crucial to determine whether 3D global strain values are superior to EFs and volumetric parameters. However, there are few commercial software packages that can incorporate 3D RV and LA strains. Fourth, although we demonstrated that 3D RVEF is better than 2D RV parameters, its accuracy and reliability depend on the expertise of the examiner. 3DE data acquisition was not always possible in every AS patient. In such situations, 2D RVFAC and RVGLS are a potential alternative. Fifth, although significant tricuspid regurgitation (TR) and mitral regurgitation (MR) have potential to affect the prognosis of patients with AS, there were not significant association with cardiac events in this study. This may be due to the small sample size in more than moderate TR and MR. Finally, this was a single-center retrospective study. To validate our concepts, a multi-center prospective study is required.

## Conclusions

3D RVEF had significant prognostic value, even after adjusting 3D left heart parameters and comorbidities in patients with asymptomatic AS. A cut-off value of RVEF ≤ 40% should be considered for better management in asymptomatic AS patients.

## Data Availability Statement

The raw data supporting the conclusions of this article will be made available by the authors, without undue reservation.

## Ethics Statement

The studies involving human participants were reviewed and approved by the Ethics Committee at the University of Occupational and Environmental Health. Written informed consent for participation was not required for this study in accordance with the national legislation and the institutional requirements.

## Author Contributions

YN: data curation, data analysis, and writing—draft. TK: data curation. MT: conceptualization, methodology, supervision, writing—review, and editing. All authors contributed to the article and approved the submitted version.

## Conflict of Interest

The authors declare that the research was conducted in the absence of any commercial or financial relationships that could be construed as a potential conflict of interest.

## Publisher's Note

All claims expressed in this article are solely those of the authors and do not necessarily represent those of their affiliated organizations, or those of the publisher, the editors and the reviewers. Any product that may be evaluated in this article, or claim that may be made by its manufacturer, is not guaranteed or endorsed by the publisher.
