# Supplementary material for: Prognostic Value of the Three-Dimensional Right Ventricular Ejection Fraction in Patients With Asymptomatic Aortic Stenosis
Source: Front Cardiovasc Med. 2021 Dec 13;8:795016. doi: 10.3389/fcvm.2021.795016 (PMC8710536; doi:10.3389/fcvm.2021.795016)
Supplement: Supplementary file 1 [file Table_1.docx]

Table S1: Multivariate Cox regression analyses involving RVFAC after adjusting Charlson index and AVR as time-dependent covariates

|  | Mean PG model | | iAVA model | | SVi model | | E/e’ model | | LAVIn model | |
| --- | --- | --- | --- | --- | --- | --- | --- | --- | --- | --- |
|  | HR (95% CI) | P value | HR (95% CI) | P value | HR (95% CI) | P value | HR (95% CI) | P value | HR (95% CI) | P value |
| LVEF | 0.919 (0.887-0.953) | <0.001 | 0.929 (0.896-0.963) | <0.001 | 0.927 (0.892-0.964) | <0.001 | 0.926 (0.893-0.961) | <0.001 | 0.963 (0.898-0.972) | <0.001 |
| RVFAC | 0.951 (0.917-0.986) | 0.007 | 0.955 (0.922-0.990) | 0.012 | 0.962 (0.928-0.997) | 0.034 | 0.963 (0.929-0.997) | 0.033 | 0.963 (0.929-0.999) | 0.043 |
| Mean PG | 1.037 (1.018-1.057) | <0.001 |  |  |  |  |  |  |  |  |
| iAVA |  |  | 0.079 (0.017-0.370) | 0.001 |  |  |  |  |  |  |
| SVi |  |  |  |  | 0.983 (0.953-1.014) | 0.279 |  |  |  |  |
| E/e’ |  |  |  |  |  |  | 1.025 (1.000-1.052) | 0.050 |  |  |
| LAVIn |  |  |  |  |  |  |  |  | 1.028 (1.013-1.044) | <0.001 |

AVR, aortic valve replacement; CI, confidence interval; HR, hazard ratio; iAVA, indexed aortic valve area; LAVIn, minimal left atrial volume index; LVEF, left ventricular ejection fraction; RVFAC, right ventricular fractional area change; SVi, stroke volume index.
