# Supplementary material for: Prognostic Value of the Three-Dimensional Right Ventricular Ejection Fraction in Patients With Asymptomatic Aortic Stenosis
Source: Front Cardiovasc Med. 2021 Dec 13;8:795016. doi: 10.3389/fcvm.2021.795016 (PMC8710536; doi:10.3389/fcvm.2021.795016)
Supplement: Supplementary file 2 [file Table_2.docx]

Table S2: Multivariate Cox regression analyses involving RVfwLS after adjusting Charlson index and AVR as time-dependent covariates

|  | Mean PG model | | iAVA model | | SVi model | | E/e’ model | | LAVIn model | |
| --- | --- | --- | --- | --- | --- | --- | --- | --- | --- | --- |
|  | HR (95% CI) | P value | HR (95% CI) | P value | HR (95% CI) | P value | HR (95% CI) | P value | HR (95% CI) | P value |
| LVEF | 0.914 (0.883-0.945) | <0.001 | 0.923 (0.892-0.956) | <0.001 | 0.923 (0.888-0.959) | <0.001 | 0.923 (0.891-0.956) | <0.001 | 0.929 (0.895-0.964) | <0.001 |
| RVfwLS | 0.935 (0.884-0.989) | 0.018 | 0.940 (0.889-0.993) | 0.027 | 0.948 (0.896-1.002) | 0.060 | 0.944 (0.893-0.997) | 0.038 | 0.950 (0.898-1.005) | 0.074 |
| Mean PG | 1.037 (1.017-1.058) | <0.001 |  |  |  |  |  |  |  |  |
| iAVA |  |  | 0.091 (0.021-0.394) | 0.001 |  |  |  |  |  |  |
| SVi |  |  |  |  | 0.982 (0.952-1.014) | 0.268 |  |  |  |  |
| E/e’ |  |  |  |  |  |  | 1.026 (1.001-1.052) | 0.039 |  |  |
| LAVIn |  |  |  |  |  |  |  |  | 1.028 (1.014-1.043) | <0.001 |

AVR, aortic valve replacement; CI, confidence interval; HR, hazard ratio; iAVA, indexed aortic valve area; LAVIn, minimal left atrial volume index; LVEF, left ventricular ejection fraction; RVfwLS, right ventricular free-wall longitudinal strain; SVi, stroke volume index.
