# Supplementary material for: Prognostic Value of the Three-Dimensional Right Ventricular Ejection Fraction in Patients With Asymptomatic Aortic Stenosis
Source: Front Cardiovasc Med. 2021 Dec 13;8:795016. doi: 10.3389/fcvm.2021.795016 (PMC8710536; doi:10.3389/fcvm.2021.795016)
Supplement: Supplementary file 3 [file Table_3.docx]

Table S3: Multivariate Cox regression analyses involving RVGLS after adjusting Charlson index and AVR as time-dependent covariates

|  | Mean PG model | | iAVA model | | SVi model | | E/e’ model | | LAVIn model | |
| --- | --- | --- | --- | --- | --- | --- | --- | --- | --- | --- |
|  | HR (95% CI) | P value | HR (95% CI) | P value | HR (95% CI) | P value | HR (95% CI) | P value | HR (95% CI) | P value |
| LVEF | 0.924 (0.892-0.957) | <0.001 | 0.934 (0.902-0.968) | <0.001 | 0.932 (0.897-0.969) | <0.001 | 0.932 (0.898-0.967) | <0.001 | 0.937 (0.902-0.973) | <0.001 |
| RVGLS | 0.873 (0.804-0.949) | 0.001 | 0.878 (0.808-0.955) | 0.002 | 0.894 (0.823-0.971) | 0.008 | 0.892 (0.822-0.968) | 0.006 | 0.904 (0.833-0.982) | 0.016 |
| Mean PG | 1.038 (1.018-1.058) | <0.001 |  |  |  |  |  |  |  |  |
| iAVA |  |  | 0.087 (0.020-0.379) | 0.001 |  |  |  |  |  |  |
| SVi |  |  |  |  | 0.985 (0.954-1.016) | 0.326 |  |  |  |  |
| E/e’ |  |  |  |  |  |  | 1.024 (0.999-1.050) | 0.064 |  |  |
| LAVIn |  |  |  |  |  |  |  |  | 1.025 (1.010-1.041) | <0.001 |

AVR, aortic valve replacement; CI, confidence interval; HR, hazard ratio; iAVA, indexed aortic valve area; LAVIn, minimal left atrial volume index; LVEF, left ventricular ejection fraction; RVGLS, right ventricular global longitudinal strain; SVi, stroke volume index.
