# Supplementary material for: Prognostic Value of the Three-Dimensional Right Ventricular Ejection Fraction in Patients With Asymptomatic Aortic Stenosis
Source: Front Cardiovasc Med. 2021 Dec 13;8:795016. doi: 10.3389/fcvm.2021.795016 (PMC8710536; doi:10.3389/fcvm.2021.795016)
Supplement: Supplementary file 4 [file Table_4.DOCX]

Table S4: Multivariate Cox regression analyses including 3D and conventional 2D RV parameters after adjusting Charlson’s comorbidity index and AVR as time-dependent covariates

|  | RVFAC model | | RVGLS model | | RVfwLS model | |
| --- | --- | --- | --- | --- | --- | --- |
|  | HR (95% CI) | P value | HR (95% CI) | P value | HR (95% CI) | P value |
| LVEF | 0.945 (0.906-0.986) | 0.009 | 0.950 (0.911-0.991) | 0.017 | 0.946 (0.907-0.986) | 0.009 |
| RVEF | 0.909 (0.872-0.948) | <0.001 | 0.917 (0.879-0.956) | <0.001 | 0.910 (0.873-0.948) | <0.001 |
| RVFAC | 1.008 (0.969-1.049) | 0.699 |  |  |  |  |
| RVGLS |  |  | 0.979 (0.893-1.073) | 0.653 |  |  |
| RVfwLS |  |  |  |  | 1.011 (0.951-1.075) | 0.726 |

2D, two-dimensional, 3D, three-dimensional; AVR, aortic valve replacement; CI, confidence interval; HR, hazard ratio; PH, pulmonary hypertension; RVEF, right ventricular ejection fraction; RVFAC, right ventricular fractional area change; RVfwLS, right ventricular free-wall longitudinal strain; RVGLS, right ventricular global longitudinal strain.
