# Supplementary material for: Prognostic Value of the Three-Dimensional Right Ventricular Ejection Fraction in Patients With Asymptomatic Aortic Stenosis
Source: Front Cardiovasc Med. 2021 Dec 13;8:795016. doi: 10.3389/fcvm.2021.795016 (PMC8710536; doi:10.3389/fcvm.2021.795016)
Supplement: Supplementary file 5 [file Table_5.docx]

Table S5: Univariate Cox regression analyses of predictors of cardiac events in patients with “more-than-moderate-to-severe” AS.

|  | HR | 95% CI | Z score | P value |
| --- | --- | --- | --- | --- |
| Age (per 1 y.o increase) | 1.014 | 0.977-1.053 | 0.730 | 0.465 |
| Sex (Male) | 1.925 | 1.017-3.642 | 2.013 | 0.044 |
| BMI (per 1 kg/m^2^ increase) | 0.952 | 0.864-1.049 | -0.988 | 0.323 |
| BSA (per 1 m^2^ increase) | 1.916 | 0.293-12.54 | 0.679 | 0.497 |
| SBP (per 1 mmHg increase) | 0.997 | 0.984-1.010 | -0.446 | 0.656 |
| DBP (per 1mmHg increase) | 1.001 | 0.979-1.023 | 0.066 | 0.947 |
| Heat Rate (per 1 bpm increase) | 1.039 | 1.015-1.064 | 3.203 | 0.001 |
| E-wave (per 1 cm/s increase) | 1.019 | 1.002-1.017 | 2.345 | 0.019 |
| E/A (per 1-unit increase) | 1.216 | 0.678-2.182 | 0.656 | 0.512 |
| E/e’ (per 1-unit increase) | 1.026 | 1.006-1.047 | 2.560 | 0.011 |
| SPAP (per 1 mmHg increase) | 1.024 | 0.994-1.056 | 1.554 | 0.120 |
| RVFAC (per 1% increase) | 0.897 | 0.859-0.937 | -4.857 | <0.001 |
| RVfwLS (per 1% increase) | 0.881 | 0.826-0.940 | -3.853 | <0.001 |
| RVGLS (per 1% increase) | 0.900 | 0.733-0.874 | -4.982 | <0.001 |
| TR=severe | 5.078 | 0.603-42.77 | 1.495 | 0.135 |
| TR=moderate | 1.532 | 0.317-7.408 | 0.531 | 0.596 |
| Peak velocity (per 1 m/s increase) | 1.154 | 0.750-1.776 | 0.649 | 0.516 |
| Mean PG (per 1 mmHg increase) | 1.013 | 0.992-1.036 | 1.196 | 0.232 |
| Indexed AVA (per 1 cm^2^/m^2^ increase) | 0.034 | 0.002-0.620 | -2.281 | 0.023 |
| SVi (per 1 mL/m^2^ increase) | 0.954 | 0.917-0.992 | -2.355 | 0.019 |
| AVR as time-dependent variable (yes) | 1.074 | 0.444-2.602 | 0.159 | 0.874 |
| Charlson’s index (per 1-point increase) | 1.287 | 1.105-1.500 | 3.241 | 0.001 |
| 3D LVEDVI (per 1 mL/m^2^ increase) | 1.030 | 1.011-1.050 | 3.139 | 0.002 |
| 3D LVESVI (per 1 mL/m^2^ increase) | 1.056 | 1.039-1.075 | 6.302 | <0.001 |
| 3D LVEF (per 1% increase) | 0.889 | 0.861-0.918 | -7.183 | <0.001 |
| 3D LVMI (per 1 g/m^2^ increase) | 1.037 | 1.022-1.052 | 2.979 | <0.001 |
| 3D LAVIx (per 1 mL/m^2^ increase) | 1.023 | 1.011-1.036 | 3.724 | <0.001 |
| 3D LAVIn (per 1 mL/m^2^ increase) | 1.031 | 1.018-1.044 | 5.508 | <0.001 |
| 3D RVEDVI (per 1 mL/m^2^ increase) | 1.028 | 1.014-1.043 | 3.912 | <0.001 |
| 3D RVESVI (per 1 mL/m^2^ increase) | 1.069 | 1.048-1.090 | 6.512 | <0.001 |
| 3D RVEF (per 1% increase) | 0.899 | 0.868-0.931 | -5.973 | <0.001 |

3D, three-dimensional; AVA, aortic valve area; AVR, aortic valve replacement; BMI, body mass index; BSA, body surface area; CI, confidence interval; DBP, diastolic blood pressure; LAEF, left atrial emptying fraction; LAVIn, minimum left atrial volume index; LAVIx, maximum left atrial volume index; LVEDVI, left ventricular end-diastolic volume index; LVEF, left ventricular ejection fraction; LVESVI, left ventricular end-systolic volume index; LVMI, left ventricular mass index; HR, hazard ratio; PG, pressure gradient; RVEDVI, right ventricular end-diastolic volume index; RVEF, right ventricular ejection fraction; RVESVI, right ventricular end-systolic volume index; RVFAC, right ventricular fractional area change; RVfwLS, right ventricular free-wall longitudinal strain; RVGLS, right ventricular global longitudinal strain; SBP, systolic blood pressure; SPAP, systolic pulmonary artery pressure; SVi, stroke volume index.
