# Supplementary material for: Prognostic Value of the Three-Dimensional Right Ventricular Ejection Fraction in Patients With Asymptomatic Aortic Stenosis
Source: Front Cardiovasc Med. 2021 Dec 13;8:795016. doi: 10.3389/fcvm.2021.795016 (PMC8710536; doi:10.3389/fcvm.2021.795016)
Supplement: Supplementary file 6 [file Table_6.docx]

Table S6: Multivariate Cox regression analyses after adjusting Charlson index and AVR as time-dependent covariates in patients with severe AS.

|  | LVEF model | | iAVA model | | LVMI model | | E/e’ model | | LAVIn model | |
| --- | --- | --- | --- | --- | --- | --- | --- | --- | --- | --- |
|  | HR (95% CI) | P value | HR (95% CI) | P value | HR (95% CI) | P value | HR (95% CI) | P value | HR (95% CI) | P value |
| RVEF | 0.936 (0.899-0.975) | 0.001 | 0.901 (0.868-0.935) | <0.001 | 0.918 (0.884-0.953) | <0.001 | 0.900 (0.868-0.934) | <0.001 | 0.915 (0.879-0.953) | <0.001 |
| LVEF | 0.922 (0.884-0.962) | <0.001 |  |  |  |  |  |  |  |  |
| iAVA |  |  | 0.011 (0.001-0.276) | 0.006 |  |  |  |  |  |  |
| LVMI |  |  |  |  | 1.024 (1.001-1.040) | 0.002 |  |  |  |  |
| E/e’ |  |  |  |  |  |  | 1.031 (1.002-1.060) | 0.032 |  |  |
| LAVIn |  |  |  |  |  |  |  |  | 1.024 (1.008-1.040) | 0.003 |

AVR, aortic valve replacement; CI, confidence interval; HR, hazard ratio; iAVA, indexed aortic valve area; LAVIn, minimal left atrial volume index; LVEF, left ventricular ejection fraction; LVMI, left ventricular mass index; RVEF, right ventricular ejection fraction.
