# Supplementary material for: Prognostic Value of the Three-Dimensional Right Ventricular Ejection Fraction in Patients With Asymptomatic Aortic Stenosis
Source: Front Cardiovasc Med. 2021 Dec 13;8:795016. doi: 10.3389/fcvm.2021.795016 (PMC8710536; doi:10.3389/fcvm.2021.795016)
Supplement: Supplementary file 7 [file Table_7.DOCX]

Table S7: Univariate Cox regression analyses of predictors of cardiac events in patients with “less-than-moderate-to-severe” AS.

|  | HR | 95% CI | Z score | P value |
| --- | --- | --- | --- | --- |
| Age (per 1 y.o increase) | 1.038 | 0.976-1.104 | 1.183 | 0.237 |
| Sex (Male) | 0.776 | 0.287-2.097 | -0.500 | 0.617 |
| BMI (per 1 kg/m^2^ increase) | 0.957 | 0.844-1.085 | -0.684 | 0.494 |
| BSA (per 1 m^2^ increase) | 0.427 | 0.038-4.730 | -0.694 | 0.488 |
| SBP (per 1 mmHg increase) | 0.986 | 0.965-1.007 | -1.312 | 0.190 |
| DBP (per 1mmHg increase) | 0.984 | 0.945-1.025 | -0.767 | 0.443 |
| Heat Rate (per 1 bpm increase) | 1.012 | 0.974-1.052 | 0.631 | 0.528 |
| E-wave (per 1 cm/s increase) | 1.013 | 0.997-1.029 | 1.546 | 0.122 |
| E/A (per 1-unit increase) | 1.613 | 0.749-3.472 | 1.223 | 0.222 |
| E/ε’ (per 1-unit increase) | 1.063 | 1.004-1.126 | 2.103 | 0.035 |
| SPAP (per 1 mmHg increase) | 0.989 | 0.929-1.052 | -0.336 | 0.714 |
| RVFAC (per 1% increase) | 0.954 | 0.891-1.022 | -1.334 | 0.182 |
| RVfwLS (per 1% increase) | 0.909 | 0.821-1.006 | -1.852 | 0.064 |
| RVGLS (per 1% increase) | 0.856 | 0.749-0.977 | -2.302 | 0.021 |
| Peak velocity (per 1 m/s increase) | 0.564 | 0.215-1.480 | -1.164 | 0.245 |
| Mean PG (per 1 mmHg increase) | 0.973 | 0.901-1.050 | -0.704 | 0.482 |
| Indexed AVA (per 1 cm^2^/m^2^ increase) | 0.031 | 0.001-1.623 | -1.720 | 0.085 |
| SVi (per 1 mL/m^2^ increase) | 0.940 | 0.887-0.996 | -2.112 | 0.035 |
| AVR as time-dependent variable (yes) | 0.603 | 0.073-4.966 | -0.470 | 0.638 |
| Charlson’s index (per 1-point increase) | 1.366 | 1.044-1.788 | 2.274 | 0.023 |
| 3D LVEDVI (per 1 mL/m^2^ increase) | 0.995 | 0.972-1.018 | -0.465 | 0.642 |
| 3D LVESVI (per 1 mL/m^2^ increase) | 1.013 | 0.985-1.041 | 0.875 | 0.382 |
| 3D LVEF (per 1% increase) | 0.916 | 0.861-0.975 | -2.743 | 0.006 |
| 3D LVMI (per 1 g/m^2^ increase) | 1.004 | 0.988-1.020 | 0.499 | 0.618 |
| 3D LAVIx (per 1 mL/m^2^ increase) | 1.014 | 0.980-1.049 | 0.793 | 0.428 |
| 3D LAVIn (per 1 mL/m^2^ increase) | 1.037 | 0.997-1.078 | 1.803 | 0.071 |
| 3D RVEDVI (per 1 mL/m^2^ increase) | 1.004 | 0.976-1.032 | 0.258 | 0.797 |
| 3D RVESVI (per 1 mL/m^2^ increase) | 1.051 | 1.013-1.090 | 2.677 | 0.007 |
| 3D RVEF (per 1% increase) | 0.863 | 0.811-0.919 | -4.622 | <0.001 |

3D, three-dimensional; AVA, aortic valve area; AVR, aortic valve replacement; BMI, body mass index; BSA, body surface area; CI, confidence interval; DBP, diastolic blood pressure; LAEF, left atrial emptying fraction; LAVIn, minimum left atrial volume index; LAVIx, maximum left atrial volume index; LVEDVI, left ventricular end-diastolic volume index; LVEF, left ventricular ejection fraction; LVESVI, left ventricular end-systolic volume index; LVMI, left ventricular mass index; HR, hazard ratio; PG, pressure gradient; RVEDVI, right ventricular end-diastolic volume index; RVEF, right ventricular ejection fraction; RVESVI, right ventricular end-systolic volume index; RVFAC, right ventricular fractional area change; RVfwLS, right ventricular free-wall longitudinal strain; RVGLS, right ventricular global longitudinal strain; SBP, systolic blood pressure; SPAP, systolic pulmonary artery pressure; SVi, stroke volume index.
