# Supplementary material for: Prognostic Value of the Three-Dimensional Right Ventricular Ejection Fraction in Patients With Asymptomatic Aortic Stenosis
Source: Front Cardiovasc Med. 2021 Dec 13;8:795016. doi: 10.3389/fcvm.2021.795016 (PMC8710536; doi:10.3389/fcvm.2021.795016)
Supplement: Supplementary file 8 [file Table_8.docx]

Table S8: Multivariate logistic regression analyses for RVEF<40% after adjusting CAD, CKD, and AF

|  | Heart rate model | | iAVA model | | LVSVi model | | LVEF model | | LAVIn model | |
| --- | --- | --- | --- | --- | --- | --- | --- | --- | --- | --- |
|  | OR (95% CI) | P value | OR (95% CI) | P value | OR (95% CI) | P value | OR (95% CI) | P value | OR (95% CI) | P value |
| CAD | 2.084 (0.998-4.351) | 0.051 | 2.048 (0.978-4.286) | 0.057 | 1.869 (0.880-3.969) | 0.104 | 1.469 (0.672-3.211) | 0.335 | 2.399 (1.130-5.092) | 0.023 |
| CKD | 4.442 (2.023-9.753) | <0.001 | 4.684 (2.135-10.27) | <0.001 | 4.490 (2.013-10.02) | <0.001 | 3.328 (1.459-7.592) | 0.004 | 3.401 (1.523-7.594) | 0.003 |
| AF | 6.109 (2.442-15.29) | <0.001 | 5.663 (2.554-14.22) | <0.001 | 3.534 (1.354-9.225) | 0.010 | 5.190 (2.001-13.46) | 0.001 | 4.074 (1.320-12.58) | 0.015 |
| Heart rate | 1.022 (0.997-1.048) | 0.091 |  |  |  |  |  |  |  |  |
| iAVA |  |  | 0.100 (0.016-0.604) | 0.012 |  |  |  |  |  |  |
| LVSVi |  |  |  |  | 0.914 (0.875-0.955) | <0.001 |  |  |  |  |
| LVEF |  |  |  |  |  |  | 0.894 (0.855-0.936) | <0.001 |  |  |
| LAVIn |  |  |  |  |  |  |  |  | 1.026 (1.000-1.052) | 0.046 |

AF, atrial fibrillation; CAD, coronary artery disease; CI, confidence interval; CKD, chronic kidney disease; iAVA, indexed aortic valve area; LVEF, left ventricular ejection fraction; LVSVi, left ventricular stroke volume index; LAVIn, minimum left atrial volume; OR, odds ratio; RVEF, right ventricular ejection fraction.
