# Supplementary figures and images for: Prognostic Value of the Three-Dimensional Right Ventricular Ejection Fraction in Patients With Asymptomatic Aortic Stenosis
Source: Front Cardiovasc Med. 2021 Dec 13;8:795016. doi: 10.3389/fcvm.2021.795016 (PMC8710536; doi:10.3389/fcvm.2021.795016)

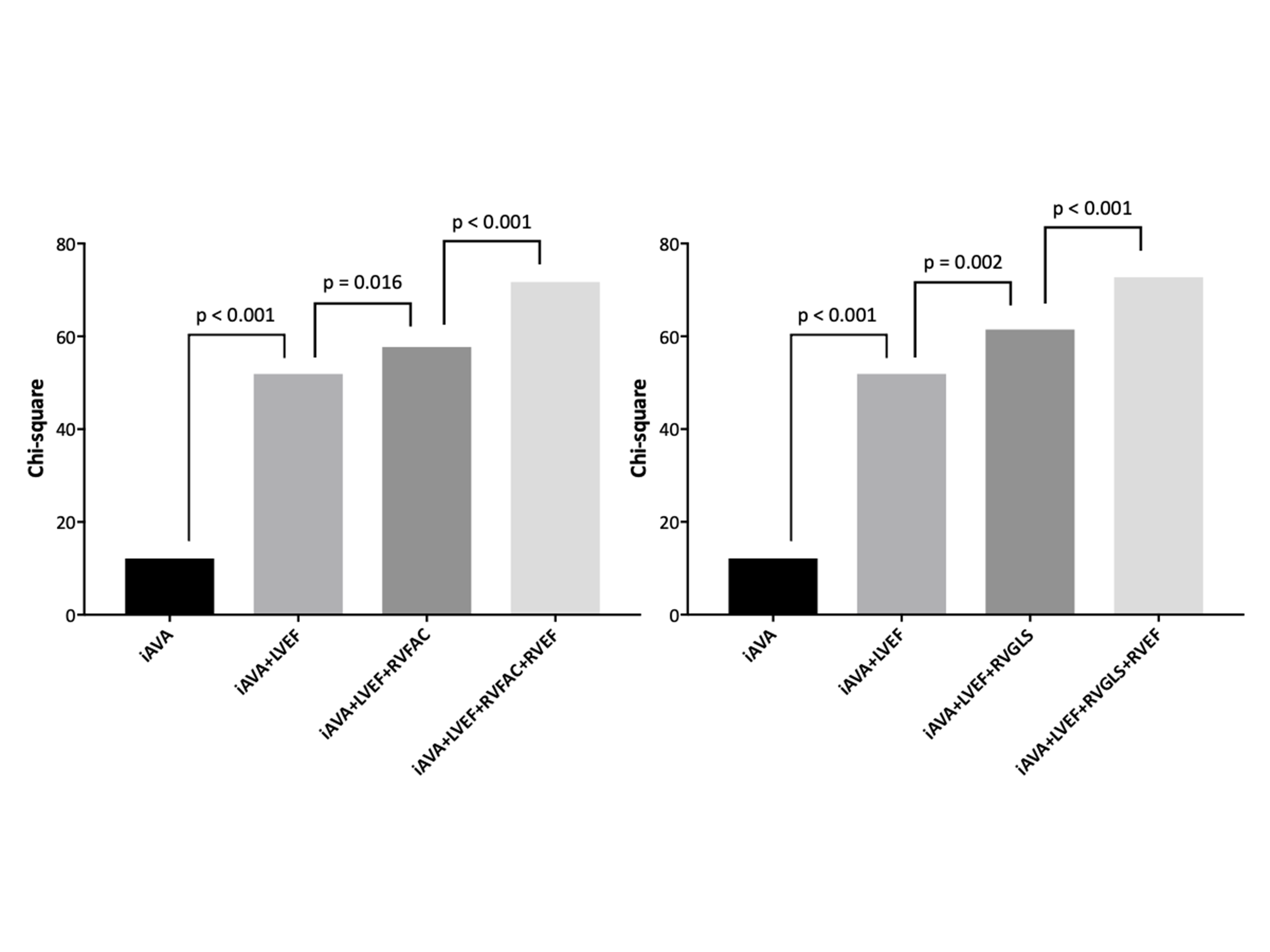

Supplement: Supplementary file 9 [file Image_1.TIFF]

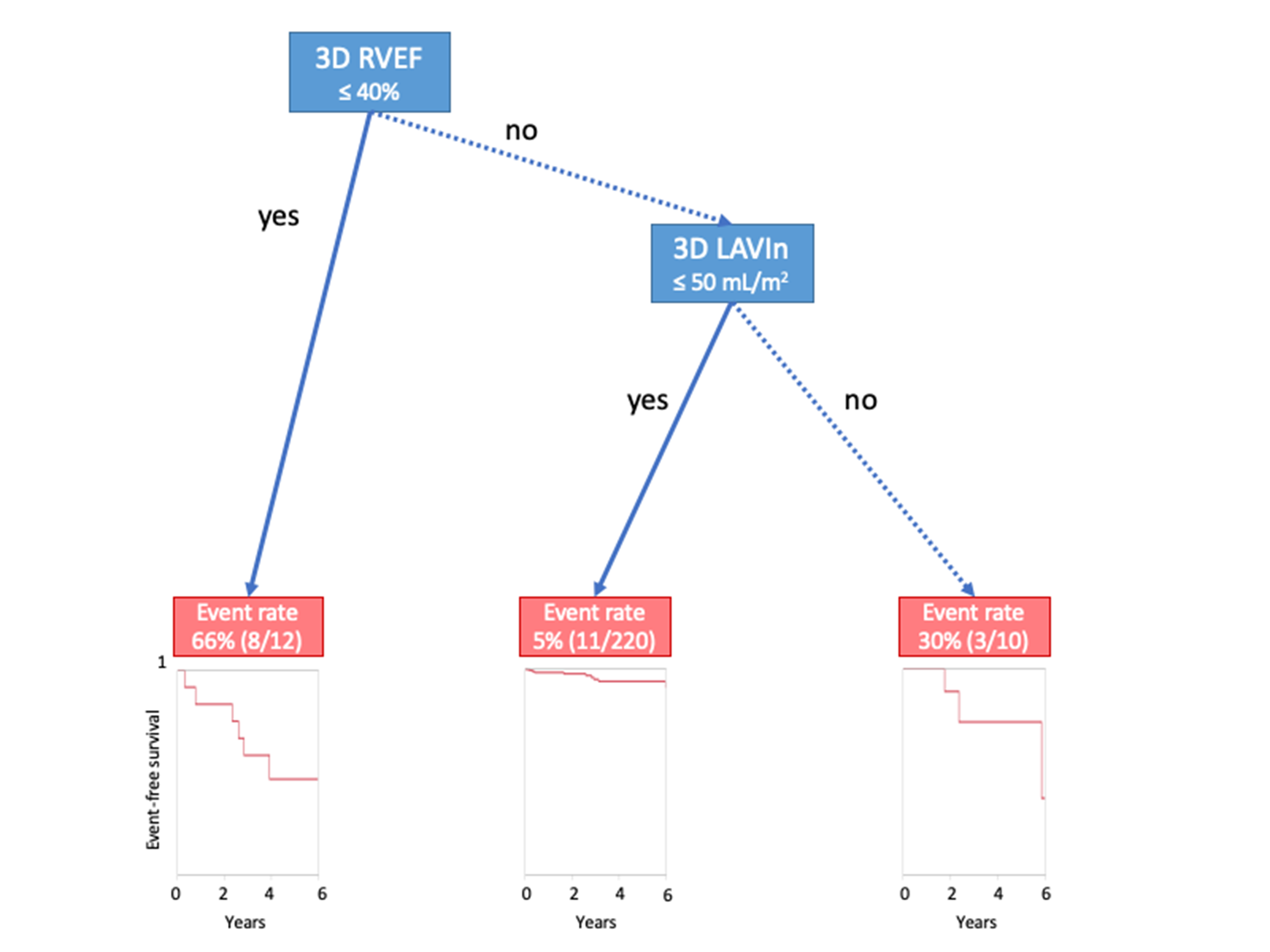

Supplement: Supplementary file 10 [file Image_2.TIFF]

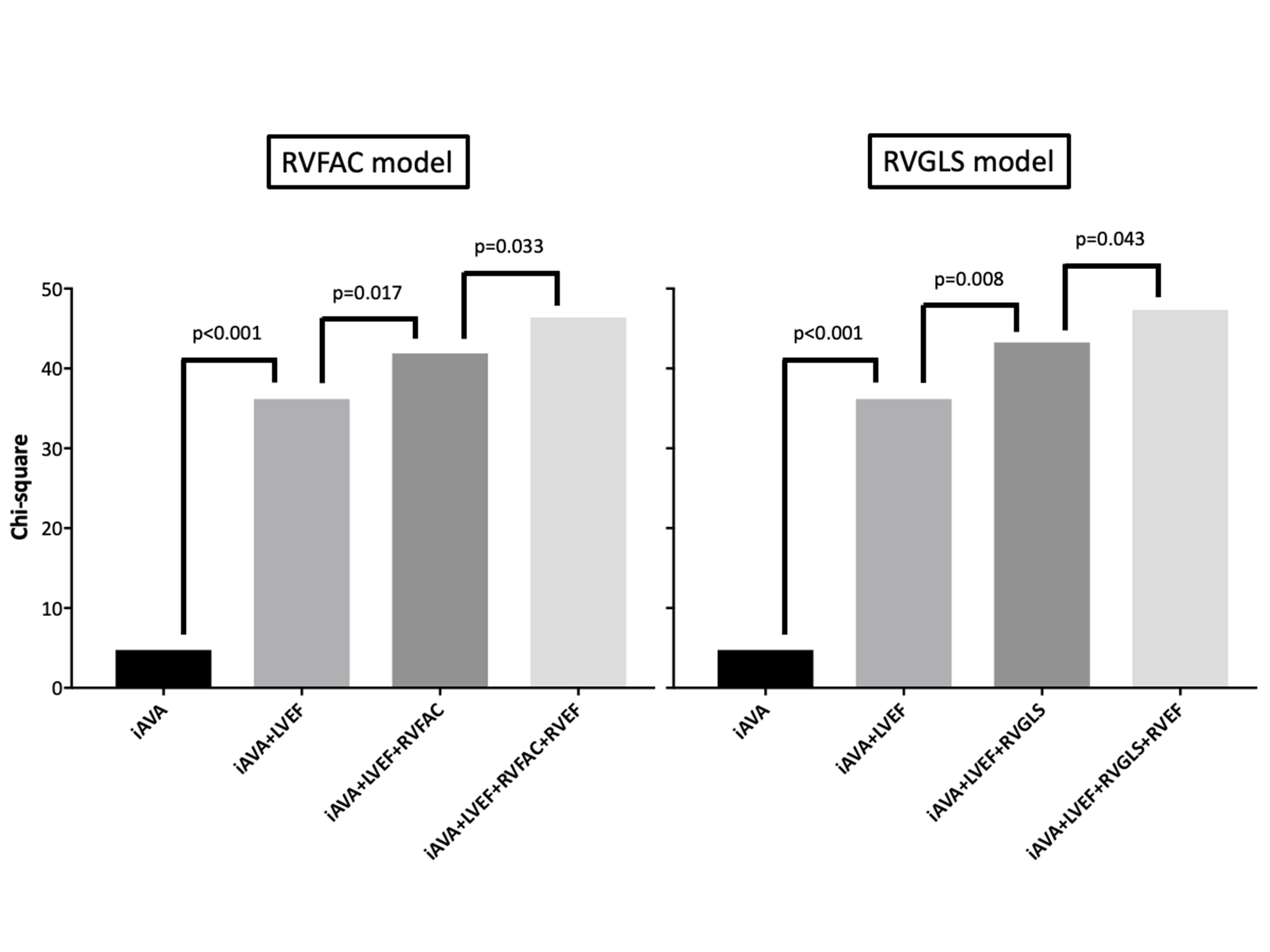

Supplement: Supplementary file 11 [file Image_3.TIFF]

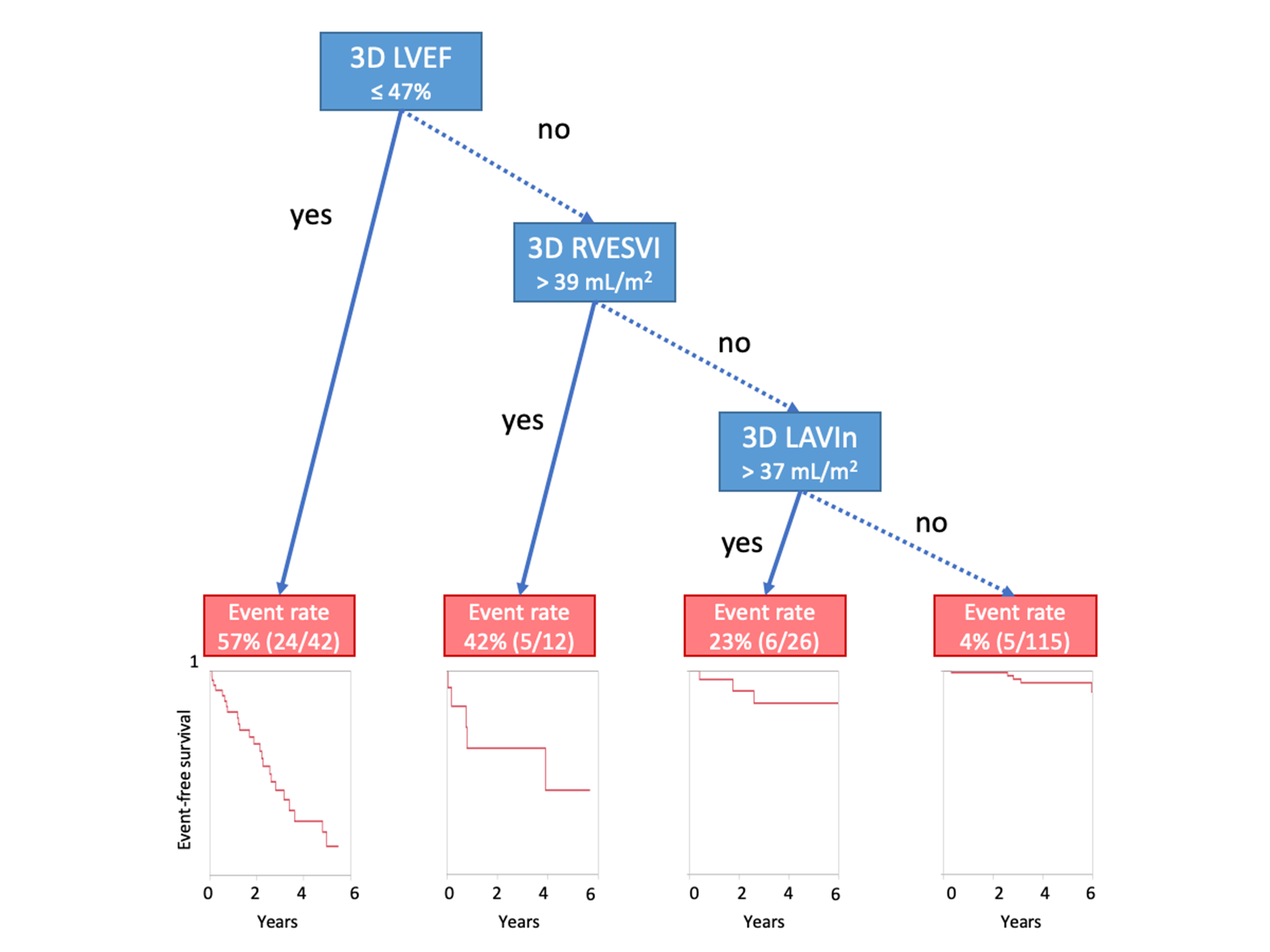

Supplement: Supplementary file 12 [file Image_4.TIFF]

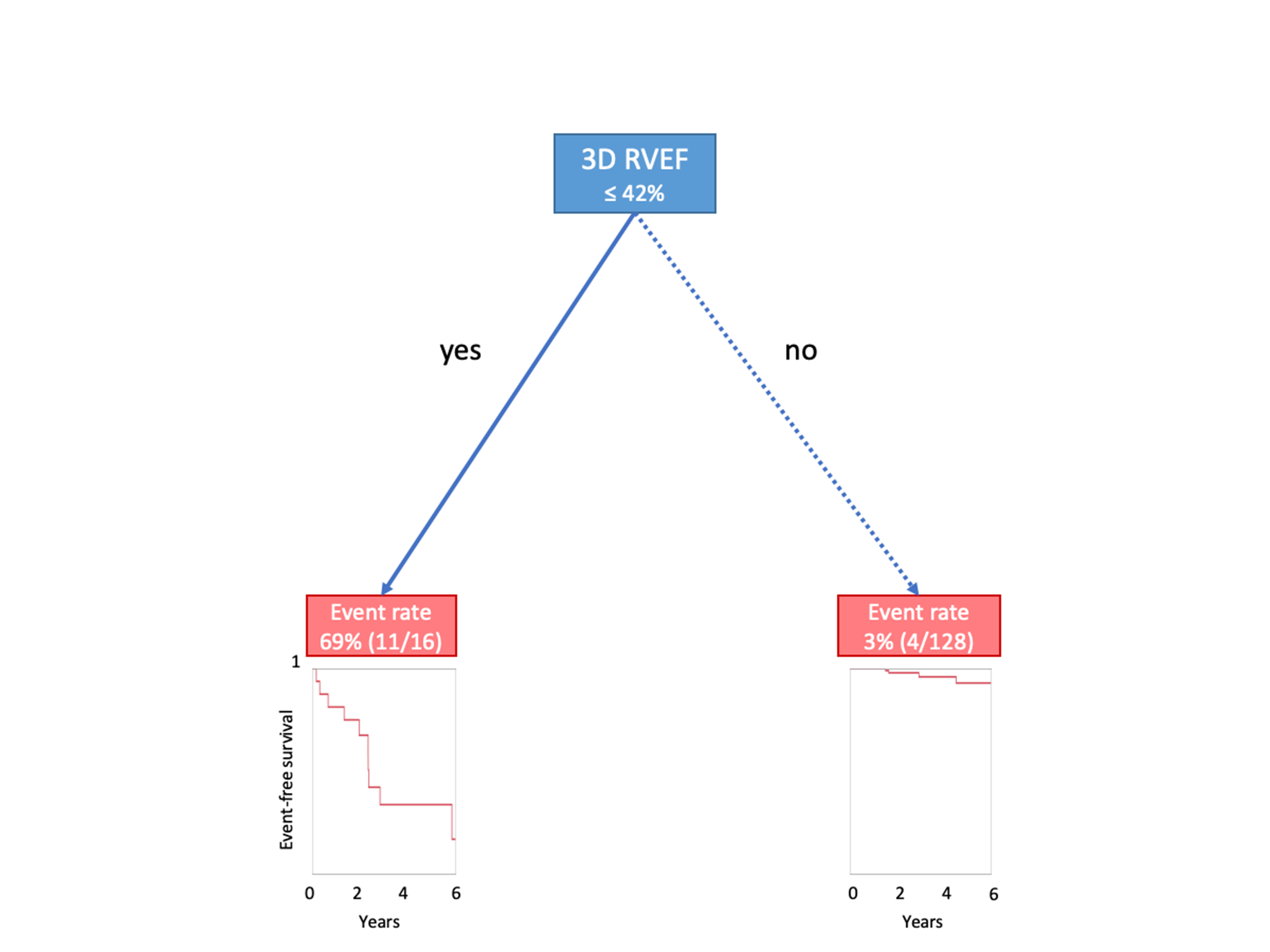

Supplement: Supplementary file 13 [file Image_5.TIFF]
